# Disease Resistance in Atlantic Salmon (*Salmo salar*): Coinfection of the Intracellular Bacterial Pathogen *Piscirickettsia salmonis* and the Sea Louse *Caligus rogercresseyi*

**DOI:** 10.1371/journal.pone.0095397

**Published:** 2014-04-15

**Authors:** Jean Paul Lhorente, José A. Gallardo, Beatriz Villanueva, María J. Carabaño, Roberto Neira

**Affiliations:** 1 Aquainnovo S.A, Puerto Montt, Chile; 2 Pontificia Universidad Católica de Valparaíso, Valparaíso, Chile; 3 Departamento de Mejora Genética Animal, INIA, Madrid, Spain; 4 Departamento de Producción Animal, Facultad de Ciencias Agronómicas, Universidad de Chile, Santiago, Chile; The Johns Hopkins University School of Medicine, United States of America

## Abstract

**Background:**

Naturally occurring coinfections of pathogens have been reported in salmonids, but their consequences on disease resistance are unclear. We hypothesized that 1) coinfection of *Caligus rogercresseyi* reduces the resistance of Atlantic salmon to *Piscirickettsia salmonis*; and 2) coinfection resistance is a heritable trait that does not correlate with resistance to a single infection.

**Methodology:**

In total, 1,634 pedigreed Atlantic salmon were exposed to a single infection (SI) of *P. salmonis* (primary pathogen) or coinfection with *C. rogercresseyi* (secondary pathogen). Low and high level of coinfection were evaluated (LC = 44 copepodites per fish; HC = 88 copepodites per fish). Survival and quantitative genetic analyses were performed to determine the resistance to the single infection and coinfections.

**Main Findings:**

*C. rogercresseyi* significantly increased the mortality in fish infected with *P. salmonis* (SI mortality = 251/545; LC mortality = 544/544 and HC mortality = 545/545). Heritability estimates for resistance to *P. salmonis* were similar and of medium magnitude in all treatments (*h* ^2^_SI_ = 0.23±0.07; *h* ^2^_LC_ = 0.17±0.08; *h* ^2^_HC_ = 0.24±0.07). A large and significant genetic correlation with regard to resistance was observed between coinfection treatments (r_g_ LC-HC = 0.99±0.01) but not between the single and coinfection treatments (*r_g_* SI-LC = −0.14±0.33; *r_g_* SI-HC = 0.32±0.34).

**Conclusions/Significance:**

*C. rogercresseyi*, as a secondary pathogen, reduces the resistance of Atlantic salmon to the pathogen *P. salmonis*. Resistance to coinfection of *Piscirickettsia salmonis* and *Caligus rogercresseyi* in Atlantic salmon is a heritable trait. The absence of a genetic correlation between resistance to a single infection and resistance to coinfection indicates that different genes control these processes. Coinfection of different pathogens and resistance to coinfection needs to be considered in future research on salmon farming, selective breeding and conservation.

## Introduction

Naturally occurring coinfections of pathogens have been reported in various salmonid species [1], [2]. However, the consequences for overall disease resistance remain unclear. Most studies have shown that presence of a primary pathogen reduces the resistance response to a secondary pathogen. For example, in rainbow trout, primary infestation of the parasite *Myxobolus cerebralis* suppresses the immune system, increasing mortality associated with a secondary infection by *Yersinia ruckeri* [3]. Similarly, Atlantic salmon infected with IPNV show significantly increased mortality as smolts when exposed to Furunculosis, Vibriosis and ISAv [4], [5]. Conversely, several studies have shown that coinfection does not necessarily increase mortality. For example, some studies on rainbow and brown trout showed that IPNV reduced the infection capacity of the hematopoietic necrosis virus (IHNV) and that the mortality of fish infected with both viruses was significantly lower than that observed in single challenges with each pathogen [6], [7], [8], [9]. Furthermore, acute coinfection of IPNv and ISAv in Atlantic salmon significantly reduced mortality compared with a single infection by ISAv [4].

Sea lice are among the most important sanitary problems in the global salmon aquaculture industry [10], further they have been linked to wild salmon and trout population declines around the world [11], [12], [13]. Salmonid infestation by sea lice may be associated with lethal or sub-lethal effects [14], [15], [16]. Sub-lethal effects may include stress [17], [18], loss of appetite, depression of the immune system and skin damage [19], [20], and therefore, it can contribute to increased susceptibility to other diseases [21], [22]. In agreement with this hypothesis, Mustafa et al. [22] reported that Atlantic salmon infected with the sea louse *Lepeophtheirus salmonis* showed increased susceptibility to a microsporidian parasite *(Loma salmonae*). Recently, Nowak et al. [23], Bustos et al. [24] and Valdes-Donoso et al. [25] suggested, based on field studies on Atlantic salmon, that sea lice affect disease resistance to the amoeba *Neoparamoeba perurans* and to ISAv.

In Chile, caligidosis caused by *Caligus rogercresseyi* and piscirickettsiosis caused by *Piscirickettsia salmonis* have historically been the most important health problems in the salmon industry in the growth-out production phase [26], [27], [28], [10]. *C. rogercresseyi* is the only sea louse that affects the Chilean salmon industry [29], and annual losses attributed to this parasite are estimated at more than $178 million US [27], [10]. *P. salmonis*, an intracellular bacterium, was described in Chile at the end of the 1980s in the X region [30], [31], [32] and was initially found to be strongly associated with Atlantic salmon. It has now extended to other salmonid species farmed in Chile [33], producing mortality rates of up to 50% in fish in the sea growing stage, with monetary losses exceeding $100 million US per year [28]. Furthermore, *P. salmonis* has been reported in different countries and can infect rainbow trout (*Oncorhynchus mykiss*), cherry salmon (*Oncorhynchus masou*), chinook salmon (*Oncorhynchus tshawytscha*) and pink salmon (*Oncorhynchus gorbuscha*) [34].

Resistance of Atlantic salmon to *C. rogercresseyi* and *P. salmonis* has recently been studied in single-infection challenges in laboratory conditions by Yañez et al. [35] and Lhorente et al. [36]. Lhorente et al. [36] reported heritability of resistance of Atlantic salmon to the sea louse *C. rogercresseyi* of low (0.03–0.06) and medium (0.22–0.34) magnitudes for the mobile and sessile stages of the parasite, respectively. Other studies performed on different sea lice species confirmed that Atlantic salmon have a heritable defensive mechanism against *Lepeophtheirus salmonis* [37], [38], [39] and *Caligus elongatus* [40]. Additionally, it has been shown that both wild and farmed Atlantic salmon have a heritable defensive mechanism against various bacteria [41], [42], [43], [44], including *P. salmonis* [45]. For instance, Yañez et al. [35] reported heritability of resistance of Atlantic salmon to *P. salmonis* ranging from 0.11 to 0.41.

In this study, we hypothesized that coinfection with the sea louse *C. rogercresseyi* reduces the resistance of Atlantic salmon to *P. salmonis* because of the well-documented stress and depression of the immune system produced by sea lice infection in salmonids. Additionally, we hypothesized that coinfection resistance is a heritable trait that does not correlate with resistance to a single infection of *P. salmonis* because salmonid defense mechanisms against bacteria and parasites are substantially different [46]. We present experimental evidence supporting both hypotheses for the interaction between Atlantic salmon, *P. salmonis* and *C. rogercresseyi*.

## Materials and Methods

### Ethics Statement

This study was carried out in in accordance with the guide to the care and use of experimental animals of the Canadian Council on Animal Care. The protocol was approved by the Bioethical committee of the Pontificia Universidad Católica de Valparaíso (N° 10/2013). The animals were anaesthetized with benzocaine prior to the various handling processes and marking. Euthanasia was performed using an overdose of anesthesia. All efforts were made to provide the best growing conditions and to minimize suffering.

### Fish

In total, 1,634 Atlantic salmon smolt fish of 103 g average body weight from 15 full-sib families (corresponding to seven paternal half-sib families) of the AquaChile Genetic Program were available for this study. The families originated from a nested mating design (15 females and seven males), in which one male fertilized the eggs of at least two females. Eggs from each family were produced during the spawning season of 2009. The fish were fed regularly with a commercial diet and individually tagged in April 2010 at an average weight of 5 g (SD = 8.0 g). Then, they were transferred as smolts in February 2011 to the Aquadvice S.A. experimental station located at the Quillaipe sector of Puerto Montt (Chile). A health check by PCR was performed prior to transfer to verify that the fish were free of viral (*IPNv* and *ISAv*) and bacterial pathogens (*P. salmonis*, *Renibacterium salmoninarum*, *Vibrio sp* and *Flavobacteria sp*). At the experimental station, the fish underwent a three-week acclimation period under seawater conditions (salinity of 33% and a temperature of 12°C).

### Experimental Design

The fish were exposed to three different infection scenarios with three replicates (tanks) to simulate single infection by *P. salmonis* and coinfection by *P. salmonis* with two different levels of infection pressure of the parasite *C. rogercresseyi*. In all coinfection’s experiments *P. salmonis* was used as primary pathogen and *C. rogercressyi* was used as a secondary pathogen. Both levels of infection pressure were established using information from previous experiments [47] and setup to experimental fish size to ensure successful but differential settlement between treatments and no mortality associated to single sea lice infection [47], [48]. Sea lice used to produce copepodites were pathogen free (*P. salmonis* and ISAv). Fish from the 15 full-sib families were equally distributed in nine tanks of 0.72 m^3^ such that the same number of fish per family was used in each of the following treatments:

#### a) Single infection (SI)

The fish were infected with *P. salmonis* using a cohabitation challenge test in an environment free of *C. rogercresseyi*.

#### b) Low pressure of coinfection (LC)

The fish were infected with *P. salmonis* using a cohabitation challenge test and then infested with *C. rogercresseyi* using a low infestation pressure of 44 copepodites per fish.

#### c) High pressure of coinfection (HC)

The fish were infected with *P. salmonis* using a cohabitation challenge test and then infested with *C. rogercresseyi* using a high infestation pressure of 88 copepodites per fish.

The cohabitation method used for the primary infection with *P. salmonis* produces a natural infestation and reduces the manipulation of experimental fish in comparison with intra-peritoneal injection. At the beginning of the challenge with *P. salmonis* (day zero), 78 fish with unknown pedigree, referred to here as infective fish, were infected by intra-peritoneal injection. A volume of 0.2 ml/fish (at 10^6,2 ^TCID/ml) of a virulent strain of *P. salmonis,* isolated from Atlantic salmon and commercially available from ADL-Diagnostic Ltda., was injected to each infective fish. The infective fish were then placed into cohabitation with tagged fish to reach an abundance of 260 fish app. per tank and a density of 42 kg/m^3^. The mortality of the infective fish was 100% at 30 days post-infection, those fish were not included in our evaluation of resistance. Resistance to *P. salmonis* was measured as a mortality trait (dead/alive). The sexes, initial weights and final weights of all cohabitant tagged fish were recorded.

Four days post-infection, a secondary infection was conducted with *C. rogercresseyi* as described by Lhorente et al. [36]. Ten infective fish per tank were used to evaluate the effective burden of the parasite. We confirmed the lack of sea lice (i.e., chalimus I) in the SI treatment and an incremental response to settlement in both coinfection treatments (LC = 29±4.8 lice per fish; HC = 60±4.8 lice per fish).

### Statistical Analysis

Kaplan-Meier survival curves were obtained using the software Survival kit v6 [49] to define the responses of Atlantic salmon to the different treatments. A chi-squared test was used to evaluate differences between treatments [49]. To estimate fixed effects and their interactions, an ANOVA analysis was conducted using a general lineal model (GLM) [50]. Mortality was defined at 30 days after infection with *P. salmonis* and treated as a normally distributed trait [43]. The GLM was:
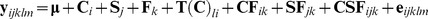
where ***y_ijklm_*** is the fish mortality condition, **μ** is the population mean, **C** ***_i_*** is the treatment effect (SI, LC or HC), **S** ***_j_*** is the sex (female or male), **F** ***_k_*** is the full sib family effect (1,2,3,…, 15), **T(C)_li_** is the l^th^ tank within the i^th^ treatment effect (C), **CF** ***_ik_*** is the treatment by family interaction, **SF** ***_jk_*** is the sex by family interaction, **CSF** ***_ijk_*** is the treatment by sex by family interaction and **e** ***_ijklm_*** is the random residual effect. The **CF** ***_ik_*** interaction determines whether families resistant to SI are also resistant to LC or HC coinfection.

A quantitative genetic analysis of the resistance to *P. salmonis* was conducted for each infection treatment to compare estimates of the additive genetic variance for the mortality trait evaluated. A threshold model (TM) was assumed to estimate variance components [51]. This model assumes a normal underlying liability variable **l** determining the categorical outcomes of the test-period survival, such that **l** *_ijk_* ≤0 corresponds to **Y** *_ijk_*_ = _0 and **l** *_ijk_* >0 corresponds to **y** *_ijk_*_ = _1. The residual variance of **l** was assumed to be 1.



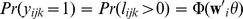
where Φ (*) corresponds to the standard normal distribution, w’*_i_* is the incidence vector that links the data with the parameters that define the mean of the distribution of the liability indexed by the parameters in θ and θ contains the population mean, the additive genetic value and the significant (P<0.05) environmental effects (sex, challenge tank, and initial weight as covariate).

Because the same families were represented in the three infection treatments (SI, LC and HC), the genetic correlation of *P. salmonis* resistance in different infective scenarios measures the interaction that determines whether families resistant to SI are also resistant to LC or HC coinfection. A set of three bivariate linear model (LM) analyses was used to estimate the genetic correlation of resistance among the different treatments:
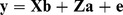
where **y** is the observations vector for the proposed traits (SI, LC or HC), **X** is the design matrix, **b** is the vector of significant (P<0.05) fixed effects within each test (sex, challenge tank and initial weight as a covariate), **Z** is the incidence matrix of the random effects, **a** is the breeding values vector and **e** is the residual error vector.

The covariance structure of random effects was:
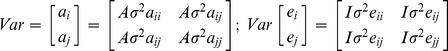



where ***a_i/j_*** and ***e_i/j_*** are the vectors of additive genetic and residual values for traits i/j, respectively; ***A*** is the additive genetic relationship matrix; ***I*** is the identify matrix; ***σ^2^a_ii/jj_*** and ***σ^2^e_ii/jj_*** are the variances of additive genetic and residual effects, respectively, for traits i/j and ***σ^2^a_ij_*** and ***σ^2^e_ij_*** are the covariances of additive genetic and residual effects, respectively, for the *ith* and *jth* traits.

Restricted Maximum Likelihood (REML) [52] and Asreml software [53] were used to solve the **TM** and **LM** models and obtain the genetic parameters.

## Results

### Development of Piscirickettsiosis with and without Coinfection with Sea Lice

**Figure 1:**
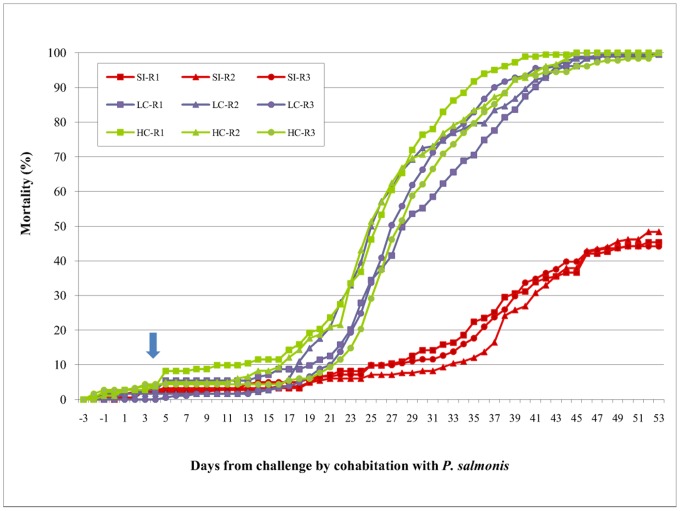
Mortality curves of Atlantic salmon coinfected with *P. salmonis* and *C. rogercresseyi*. The data show the cumulative mortality of three replicates (tanks) (R1–R3) for three different scenarios: single infection (SI) with *P. salmonis* and co-infection with two levels of infestation pressure of *C. rogercresseyi* (low pressure of coinfestation (LC) = 44 copepodites per fish; high pressure of coinfestation (HC) = 88 copepodites per fish). Each replicate had approximately 182 pedigreed fish that were free of disease. The arrow indicates the day of coinfection.

The development of piscirickettsiosis was continuously recorded over 53 days until mortality reached 544/544 (100%) and 545/545 (100%) in both coinfection treatments (Figure 1). At that time, mortality in the group that received the single infection with *P. salmonis* only reached 251/545 (46%). At the beginning of the cohabitation challenge with *P. salmonis*, a small increase in mortality was observed (day 4). This increase was likely a consequence of the sea lice infection procedure and not a direct consequence of coinfection. At 14–16 days post-infection, mortality associated with piscirickettsiosis was observed in cohabitant fish, and this result was confirmed by PCR at the beginning of the outbreak in dead fish. The daily mortality rate was lower in single than in coinfection scenarios (Figure 1), but no differences were observed between the coinfection treatments.

**Figure 2:**
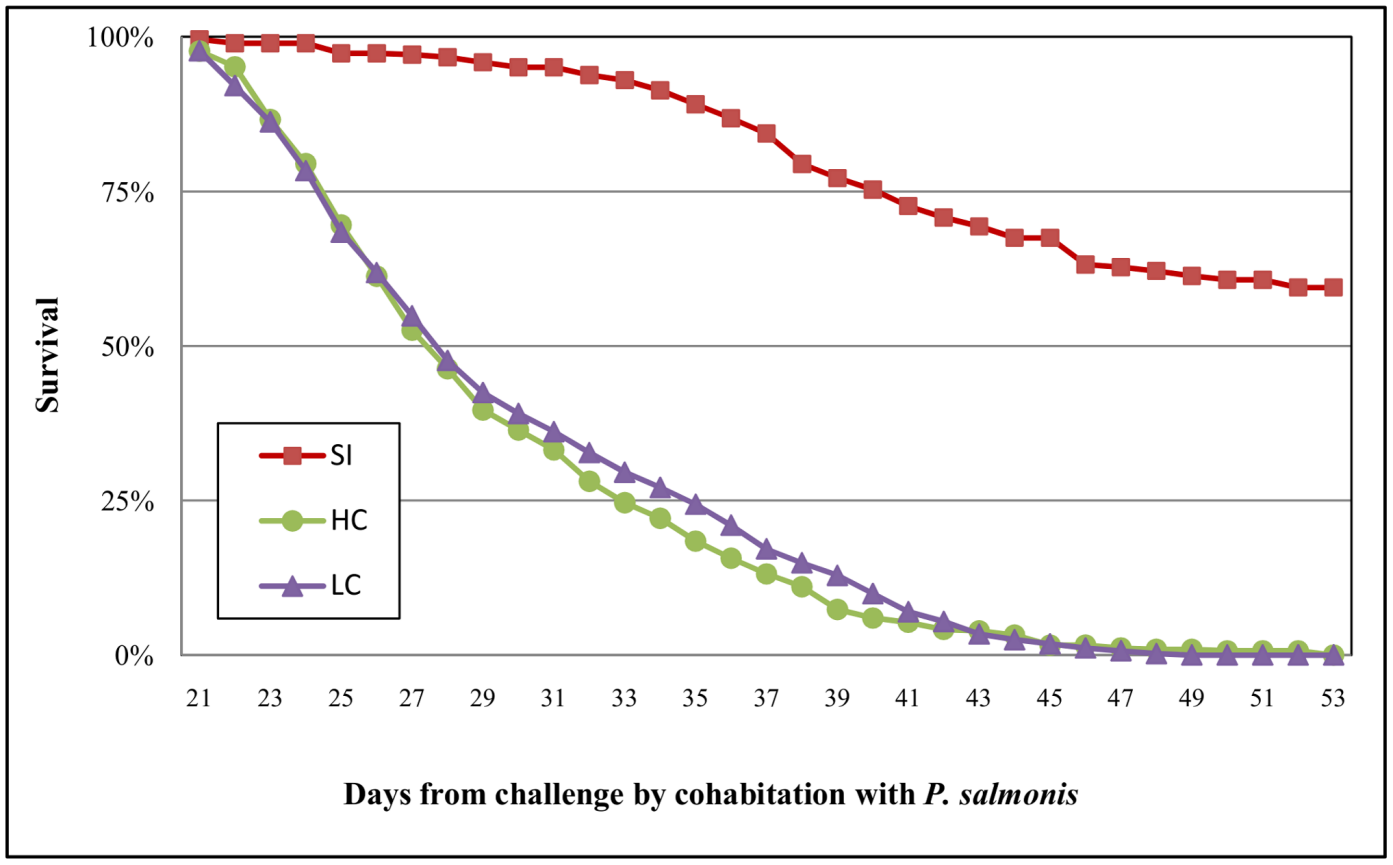
Kaplan–Meier survival function of Atlantic salmon coinfected with *P. salmonis* and *C. rogercresseyi*. The survival function represents the resistance of the Atlantic salmon (i.e., the proportion that had not died on each day following challenge) in three different treatments: single infection (SI) with *P. salmonis* and coinfection with two levels of infestation pressure of the sea louse *C. rogercresseyi* (low pressure of coinfection (LC) = 44 copepodites per fish; high pressure of co infestation (HC) = 88 copepodites per fish).

Consistent with the mortality pattern previously described, Kaplan-Meier survival curves (Figure 2) confirmed that coinfection of *P. salmonis* and *C. rogercresseyi* significantly (p<0.05) reduced the survival of Atlantic salmon compared to single infection with *P. salmonis*. However, high parasite burden did not significantly reduce the survival of Atlantic salmon compared to low parasite burden (P>0.05).

### Genetic Resistance of Atlantic Salmon to Single and Coinfection

As shown in Table 1, all main fixed effects significantly affected the mortality of Atlantic salmon challenged with *P. salmonis* and coinfected with *C. rogercresseyi*. The infection effect (I) was highly significant (P<0.001) and showed the highest relative value of the associated sum of squares over the total variability of mortality (28%). The effect of the full-sib family (F) and its interaction with the treatment effect (F×I) were also highly significant (P<0.001). These effects showed a relative influence of 7% and 4%, respectively, on the variability of mortality. Sex and tank within infection treatment effects also significantly affected mortality. However, their contributions to the overall variability of mortality were less than 2%.

**Table 1:** ANOVA results for mortality 30 days after *P. salmonis* infection.

Source of variation	fd	SS	SS%	MS	F	P-value
Infection treatment (I)	2	87.90	28.0%	43.940	310.29	0.0001
Sex (S)	1	1.75	0.6%	1.750	12.38	0.0004
Full-sib Family (F)	14	22.30	7.1%	1.590	11.25	0.0001
Tank within I (T)	6	3.72	1.2%	0.620	4.38	0.0002
I×F	28	12.94	4.1%	0.460	3.26	0.0001
S×F	2	0.53	0.2%	0.260	1.87	0.1547
S×I×F	42	6.37	2.0%	0.150	1.07	0.3517
Error	1267	179.46	57.0%			
Total	1362	314.98				

Quantitative genetic parameters for the resistance of Atlantic salmon to *P. salmonis* for the three treatments of single and coinfection are shown in Table 2. The heritability of resistance was very similar between treatments and of medium magnitude (0.17–0.24). Resistance to the single infection with *P. salmonis* did not correlate phenotypically or genetically with resistance to *P. salmonis* upon coinfection with the sea louse *C. rogercresseyi* (Table 2). Conversely, a high and significant genetic correlation for resistance to *P. salmonis* was observed between the two coinfection treatments (r*_g_* LC-HC = 0.99±0.01), providing solid evidence that these resistance values are measurements of the same trait.

**Table 2:** Estimates of heritability (on diagonal), phenotypic (above diagonal) and genetic (below diagonal) correlations (± SE) in resistance of Atlantic salmon between single infection (SI) with *P. salmonis*, and co-infection with two incremental levels the of sea louse *C. rogercresseyi* (low pressure of coinfestation (LC) = 44 copepodites per fish; high pressure of coinfestation (HC) = 88 copepodites per fish).

	SI	LC	HC
SI	0.23±0.07*	−0.04±0.09^ns^	0.06±0.07^ns^
LC	−0.14±0.33^ns^	0.17±0.08*	0.21±0.01*
HC	0.32±0.34^ns^	0.99±0.01*	0.24±0.07*

ns: Not significantly different from zero, p>0.05; *; significantly different from zero, p<0.05.

## Discussion

We have demonstrated for the first time that the sea louse *C. rogercresseyi*, as a secondary pathogen, significantly reduces the resistance of Atlantic salmon to the bacterium *P. salmonis.* The prevalence of *C. rogercresseyi* in Chilean salmon farms approaches 100% in some seasons (i.e., spring and summer) and geographic regions [27], [54]. Therefore, its effect on Atlantic salmon mortality may be higher than previously thought. In Atlantic salmon, coinfections of sea lice and other pathogens such as the amoeba *Neoparamoeba perurans* have also been reported in the USA [23] and in Chilean salmon farms [24]. Both of these studies suggested that sea lice may play an important role in the epidemiology of amoebic gill disease caused by *Neoparamoeba perurans* and/or in mortality of Atlantic salmon in sea farms. Similarly, Valdes-Donoso et al. [25] reported that most of the ISAV outbreaks between 2007 and 2009 in the X^th^ region of Chile were associated with high sea lice burdens. The reduced survival upon coinfection in Atlantic salmon might be explained by the direct skin damage caused by parasites that allows other pathogens to enter the fish [55]. Alternatively, it may result from the systemic effects of immunosuppression caused by sea lice [56].

Genetic variation in resistance to disease in salmonids has been reported for single infections of pathogens in Atlantic salmon [40], [57], [58], [36], [42], [35], rainbow trout [59], [60], [61], [62], [63], [64], Coho salmon [41] and brook charr [42]. However, genetic variation in the resistance to coinfection by two pathogens has not been previously estimated in salmonids. Using data from a farmed population of Atlantic salmon, we demonstrated genetic variation for resistance to *P. salmonis* upon coinfection with the sea louse *C. rogercresseyi*. Sea lice infections have been reported in salmon farms around the world [10], but coinfection with bacteria, viruses or parasites has been minimally investigated. Resistance to coinfection has two important implications for salmon breeding. First, if coinfection is common, selection for disease resistance to two or more pathogens, evaluated independently as proposed by Ødegård et al. [65], could be an inefficient method unless resistance to single and coinfection is positively correlated. Second, evaluation of resistance to two different pathogens could be performed in a simple assay, reducing costs associated with laboratory testing. Further studies are necessary to determine whether resistance to coinfection by *P. salmonis* and *C. rogercresseyi* or to coinfection by other pathogens relevant to salmon farming such as ISAV, *Aeromonas salmonicida* or *Neoparamoeba perurans* occurs in other populations of salmonids.

The genetic correlation for resistance among various Atlantic salmon pathogens has been described for some bacteria [66], [67], [68], [69], [70], [71] and viruses [69], [71], [72]. However, the genetic correlation for resistance between single and coinfection of two pathogens has not been previously estimated in salmonids. Our results strongly suggest that the resistance of Atlantic salmon to a single infection of *P. salmonis* and that to coinfection with the sea louse *C. rogercresseyi* are not genetically related. Therefore, we can infer that the best strategy for developing resistance to *P. salmonis* should consider coinfection with sea lice. However, further studies are necessary to establish whether the resistance to coinfection observed in experimental conditions correlates with higher survival rates in the field. A high genetic correlation for resistance between fresh and sea water has been described for other diseases such as furunculosis, sea lice and IPN [67], [38], [73].

## Conclusion

Infection with the sea louse *C. rogercresseyi*, as a secondary pathogen, reduces the resistance of Atlantic salmon to the pathogen *P. salmonis*. Resistance to coinfection of *Piscirickettsia salmonis* and *Caligus rogercresseyi* in Atlantic salmon is a heritable trait. The absence of a genetic correlation between the resistance to single infection and that to coinfection indicates that different genes control these processes. Further studies are necessary to investigate the effects of coinfection when the sea louse is the primary pathogen. It is clear that coinfection of different pathogens and resistance to coinfection needs to be considered in future research on salmon farming, selective breeding and conservation.
